# Advances in Genetics and Epigenetics of Developmental Coordination Disorder in Children

**DOI:** 10.3390/brainsci13060940

**Published:** 2023-06-11

**Authors:** Haizhen You, Junyao Shi, Fangfang Huang, Zhiyun Wei, Gary Jones, Wenchong Du, Jing Hua

**Affiliations:** 1 Department of Women and Children’s Health Care, Shanghai First Maternity and Infant Hospital, Tongji University School of Medicine, Shanghai 200120, China; 2 Women and Children Health Care Institution of Pudong District, Shanghai 200021, China; 3 NTU Psychology, School of Social Sciences, Nottingham Trent University, Nottingham NG1 6AA, UK

**Keywords:** developmental coordination disorder (DCD), genetics, co-occurrence

## Abstract

Developmental coordination disorder (DCD) is a developmental disorder characterized by impaired motor coordination, often co-occurring with attention deficit disorder, autism spectrum disorders, and other psychological and behavioural conditions. The aetiology of DCD is believed to involve brain changes and environmental factors, with genetics also playing a role in its pathogenesis. Recent research has identified several candidate genes and genetic factors associated with motor impairment, including deletions, copy number variations, single nucleotide polymorphisms, and epigenetic modifications. This review provides an overview of the current knowledge in genetic research on DCD, highlighting the importance of continued research into the underlying genetic mechanisms. While evidence suggests a genetic contribution to DCD, the evidence is still in its early stages, and much of the current evidence is based on studies of co-occurring conditions. Further research to better understand the genetic basis of DCD could have important implications for diagnosis, treatment, and our understanding of the condition’s aetiology.

## 1. Introduction

Developmental coordination disorder (DCD) is a neurodevelopmental disorder characterized by difficulties in the execution and coordination of body movements which cannot be accounted for in terms of intellectual impairment or of identifiable physical or neurological disorder [1,2]. Children with DCD display difficulties with fine and/or gross body movements such as handwriting and riding a bicycle, and are often observed frequently tripping and bumping into things [1]. Such movement difficulties have a negative impact on everyday life [3,4], and the social life and well-being of parents of children with DCD [5]. Studies have shown that 5–6% of school-aged children are diagnosed with the condition depending on the selection criteria used [3], and subtypes of DCD may exist [6]. DCD is often accompanied by other neurodevelopmental disorders including attention-deficit/hyperactivity disorder (ADHD) and autism spectrum disorder (ASD). The co-occurrence rates vary between studies; it is estimated that children with DCD have a 30–50% chance of having ADHD [7,8], and a 14–50% chance of having ASD [1,9,10]. While certain risk factors for developmental coordination disorder (DCD), such as preterm birth and male sex, have been identified [11], it is worth noting that some children diagnosed with DCD do not exhibit these conventional risk factors. This observation raises the possibility that a genetic component may underlie their motor impairment [12,13,14,15].

**Figure 1:**
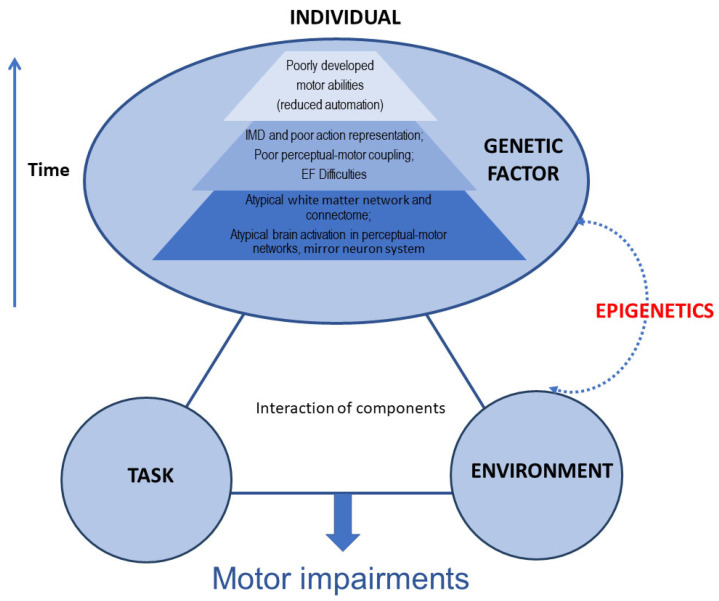
A multi-component account of developmental coordination disorder (DCD) (adjusted from Blank et al. [2]). IMD: internal modelling deficit; EF: executive function.

The mechanisms involved in DCD are not fully understood and are thought to be multifactorial. To date, advances in understanding the mechanism of DCD have been relatively limited. Much of the literature focuses on the brain changes associated with DCD, including changes in brain function and brain structure (Figure 1). For example, research has demonstrated that children with DCD have significant brain differences in motor and sensorimotor white matter pathways and has suggested that axonal development in DCD may be disrupted in this neurodevelopmental disorder [16]. There are also preliminary findings that children with DCD have smaller brain volumes within the pallidum [17]. In terms of brain function, Van Dyck et al. observed the brain resting-state functional connectivity (rsFC) of children with DCD. Their findings suggest that children with DCD exhibited increased rsFC and that this was mostly found in the dorsal extrastriate visual brain system and the cerebellum [18]. Reynolds et al. demonstrated that children with DCD had deficits supportive of the mirror neuron system (MNS) dysfunction hypothesis at a behavioural level using fMRI. Their findings also suggest that decreased activation of the thalamus, caudate, and posterior cingulate regions is associated with motor planning and attentional processes [19]. Lust et al., using sensitive electroencephalography (EEG)-based measures of MNS activation during action observation, showed that MNS function is disrupted in children with DCD [20].

In recent years, advances in gene detection technologies have led to the gradual discovery of the genome sequence and gene expression profiles associated with neurodevelopmental disorders. Although the number of studies on the genetics of DCD is limited, researchers have examined genetic factors associated with DCD, as well as genes linked with genetic susceptibility to DCD and related disorders. These studies have identified chromosome deletions, copy number variations, single nucleotide polymorphisms, and epigenetic factors that may be related to DCD. However, it should be noted that these studies are limited by relatively small patient numbers and also require function investigations to prove causation. Additionally, despite international diagnosis criteria for defining DCD [1,2], many published studies have not applied these definitions of DCD. Moreover, the evidence for a genetic contribution to DCD is largely based on studies of co-occurring conditions, and it is important to differentiate between the genetics of co-occurring conditions and the genetics of DCD itself, and that further research is needed to investigate the specific genetic mechanisms underlying DCD. Furthermore, as a complex disorder with multiple subtypes and aetiologies, it is unlikely that DCD has a single genetic mechanism that can account for its development. Nonetheless, investigating the genetic basis of DCD may provide valuable insights into the underlying neurobiological pathways that contribute to the disorder. Similarly, research on related conditions such as ASD and cerebral palsy (CP) has shown that studying individually rare mutations can illuminate the collective genetic factors underlying common disorders [21,22].

In this review, we mainly focus on the current genetic progress of DCD, as well as its co-occurrence with ADHD and ASD. Additionally, we discuss the potential environmental and epigenetic factors involved in DCD and their potential role in its development.

## 2. The Genetics of Motor Impairments

The mild-to-moderate motor impairments observed in DCD represent some of the earliest and most visible signs among affected developmental functions in neurodevelopmental disorders [23,24,25,26]. With recent technological advances, many gene-based syndromes have been identified that provide ways to reduce phenotypic heterogeneity [27,28]. For example, chromosome 22Q11.2 deletion syndrome (22q11.2 DS) is a genetic disorder caused by the deletion of a specific region on chromosome 22, with an estimated incidence of 1 in every 2000 to 4000 new-borns [29]. In addition to congenital heart disease and immunodeficiency [30], 22q11.2 DS is also associated with a high incidence of mental disorders, including ADHD, ASD, emotional disorders, and schizophrenia in adulthood [31]. Studies have found that children with 22q11.2 DS often have motor coordination impairments, including balance, hand–eye coordination, and visual motor ability. Specifically, a high percentage (81.4%) of children with 22q11.2 DS had indicative DCD (57/70). Furthermore, nine children with indicative DCD were assessed via the MABC-2 and eight of them met the DSM-5 criteria for DCD [1]. In addition, all children with 22q11.2 DS and ADHD had indicative DCD (20 of 20) [32]. 22q11.2 DS children also exhibit atypical sensorimotor control behaviours, which may contribute to the development of DCD [33]. Therefore, clinicians should be aware of the mental disorders and cognitive defects related to DCD in children with 22q11.2 DS. Moreover, this evidence suggests the implication of genetic elements within 22q11.2 in motor coordination.

Klinefelter (*47*, *XXY*) and (*48*, *XXYY*) syndromes are genetic disorders found in males, characterized by additional sex chromosomes compared with the typical male karyotype of *46*, *XY*. Both conditions have been previously associated with delays in motor development and deficits in motor skills; indeed, motor coordination impairments are common in these syndromes [34,35]. Martin et al. found that 39% of males with *XXY* and 73% of males with *XXYY* had below average visual motor integration (VMI) scores and males with *XXYY* had lower average scores compared with males with *XXY* in the domains of manual coordination, body coordination, and strength/agility measured with the BOT-2 assessments. Furthermore, fine motor dexterity and coordination deficits are also common. In order to evaluate the contributors to VMI skills, this study also supplemented tests of visual perception and motor coordination. The results show that these individuals had strengths in visual perceptual skills, which suggested that motor coordination deficits may be the predominant contributor to VMI deficits. Therefore, such motor phenotypes in *XXY* and *XXYY* should be noted for clinical practice, in order to aid in diagnosis and to guide intervention [35].

Terminal deletion of the long arm of chromosome 6 (associated with developmental delay) may also impact motor coordination. Engwerda et al. found that the *6q14.2–q14.3* region had the most influential phenotype on neurodevelopment in rare *6q* deletions in the region of *6q11* to *6q15*. The majority of individuals over the age of 2 years with a deletion in this region had moderate-to-severe developmental delay [36]. Additionally, autism spectrum disorders are frequently related to 6q deletion and an increasing number of genes in *6q* have been associated with this behavioural phenotype. A patient with a *6q11* to *6q15* deletion was found to have severe developmental delay and autism, indicating that autism may be part of the *SYNCRIP*-related phenotype [37]. Children with a *6q25* deletion spanning 11.1 Mb and 108 genes have also been found to suffer from motor impairment [38,39,40]. Their speech characterization showed elements of both childhood apraxia of speech and dysarthria due to deficits in motor coordination and low muscle tone, respectively. The overlapping deletion of the *IGF2R–AIRN–SLC22A2–SLC22A3* gene cluster in both patients is possibly associated with motor discoordination and hypotonia across multiple motor systems as well as language delays, though other genes in the deletion region may also be associated with hypotonia [38]. The *6q25* microdeletion region should be considered in the differential clinical phenotype and chromosomal microarray analysis should be performed to confirm the phenotype.

In a longitudinal study that included 56 children aged between 6 months and 8 years with *16p11.2BP4-BP5* deletion or duplication (33 deletion and 23 duplication carriers), poor motor skills and nonverbal cognitive ability were observed in 22 children with *16p11.2* deletion who were diagnosed with DCD at 24 months. Language development, however, was affected only in carriers with a duplication. These findings underscore the importance of monitoring the motor developmental trajectory of children with *16p11.2* deletion to provide personalized treatment [41]. The *Chd6* gene has been found to have high levels of expression in the brain of mice. Lathrop et al. have shown that the deletion of exon 12 of this gene led to motor coordination problems in mouse models, after excluding muscle weakness or bradykinesia and brain morphology issues [42].

To better understand the molecular basis of developmental disorders, studies have increasingly been using genomics and transcriptomics. For example, Werling et al. used RNA sequencing to identify genes related to the developmental trajectory of the human cortex, as well as common variations altering gene expression. They collected 176 dorsolateral prefrontal cortex samples from 6 gestational weeks to early adulthood (20 years old). Their analysis revealed that higher expression of *RHEBL1* was associated with the genes related to increased educational attainment [43]. However, to obtain a more comprehensive account of the molecular mechanisms underlying normal and abnormal development, it is necessary to acquire higher-resolution datasets throughout development, such as single cells, additional brain regions, and larger sample sizes. Additionally, complementary analyses of the brains of individuals with neuropsychiatric and rare genetic disorders are required. By carefully evaluating clinical and dysmorphological features, and comparing cases with overlapping rearrangements, we can begin to ascribe specific symptoms to different copy number variations (CNVs). Ultimately, this research may lead to new insights and interventions for individuals with developmental disorders.

## 3. The Genetics of DCD Co-Occurring with ADHD

Given the high co-occurrence between DCD and ADHD and other neurodevelopmental disorders, it may be beneficial to assess the genetic reasons for different neurodevelopmental disorders in individuals who present with symptoms of either disorder. In the literature, *47*, *XXY*—a chromosomal abnormality that occurs in 1 out of 650 male births—has received increased attention in recent years [44,45]. In a case report involving an 11-year-old boy diagnosed with ADHD, conventional G-banding karyotyping revealed a *47*, *XYY* karyotype. Subsequent array comparative genomic hybridization (aCGH) analysis identified an additional duplication and two deletions, each of which were associated with speech and language delay and behavioural symptoms. Specifically, the *20q13.33* deletion was linked to autism and early onset schizophrenia, while the *11p15.5* microdeletion was associated with developmental delay, autism, and epilepsy [46]. These findings underscore the importance of considering other micro-deletions, duplications, and genetic syndromes that may present with similar symptoms and of making appropriate differential diagnoses.

Moreover, recent evidence suggests that attention and motor disorders may share a common neural substrate. To investigate this relationship, Albajara et al. conducted a study examining the relationship between Developmental Coordination Disorder Questionnaire (DCDQ) scores and regional brain volumes in six regions (pre-central gyrus, post-central gyrus, inferior parietal cortex, superior frontal gyrus, middle frontal gyrus, and medial frontal gyrus) in children with ASD, ADHD, and typically developing (TD) children. Their results show that, in children with ADHD, motor impairment was associated with increased grey matter volume in the right superior frontal gyrus [47]. This unique characteristic of concurrent DCD and ADHD has the potential to improve diagnostic definitions and offer insights into the shared and separate genetic and environmental causes of attention and motor disorders.

It is worth noting that children with ADHD frequently encounter issues with motor coordination. Fliers et al. conducted genetic association testing of children with ADHD from the International Multicentre ADHD Genetic (IMAGE) cohort and found that none of the findings reached genome-wide significance. However, further bioinformatics analysis of the top-ranked findings indicated that intergenic *SNP rs11002745* on chromosome 10 and *SNP rs2839083* located 18.7 kb downstream of the *COL6A1* gene on chromosome 21, showed significant association after multi-test correction. The former SNP was correlated with gross motor problems, while the latter was correlated with fine motor problems and control in sports [48]. This study showed that eight of the top nine protein species played a role in the signal network of neural development. Enrichment analysis showed that the genes of motor neuropathy and amyotrophic lateral sclerosis were abundant, and the genes related to neurite growth and muscle function were also enriched. It has been speculated that the problem of motor coordination might be related to the genes expressed in nerve tissue and skeletal muscle. The findings of this study provide a genetic clue for the aetiology of ADHD and its associated motor coordination problems and point to a direction for the genetic research of DCD associated with ADHD. More recently, Mountford and colleagues conducted a population-based study examining the genetic association of quantitative motor coordination in children. Their findings indicate that no single nucleotide polymorphisms (SNPs) reached the level of genome-wide significance. However, they identified three chromosome regions (*3p25.2*, *6p12.1*, and *14q24.2*) that contained multiple SNPs that were suggestively associated with motor coordination. This study highlights the need for further research to better understand the genetic basis of motor coordination and related disorders [49]. To further investigate the potential shared genetic and environmental factors underlying DCD and ADHD, future studies could focus on analysing the specific genes and environmental factors that are implicated in both disorders. Additionally, studies could explore the potential interactions between these factors and how they may impact the development and manifestation of DCD and ADHD.

## 4. The Genetics of DCD Co-Occurring with ASD and Other Neurodevelopmental Disorders

Individuals with autism spectrum disorders (ASD) often experience motor problems [23,50,51,52]. One of the most well-investigated aetiologies of ASD is deletion or duplication in the *16p11.2 BP4* and *BP5* region, a recurrent ~600kb copy number variant (CNV) with an incidence of approximately 1 in 2000 (deletion) and 1 in 1100 (duplication) [33]. The neurobehavioral profile of children with the *16p11.2* deletion is characterized by pervasive speech and language impairment (>70%) and motor coordination difficulties (~60%), with 20–25% also experiencing autistic and other behavioural/psychiatric issues. Future studies are underway to expand the cohort of *16p11.2* deletion and duplication carriers, following them longitudinally over time [53]. Comprehensive clinical examinations of individuals with the *1q21.1* deletion or duplication have shown that psychiatric, neurological, and medical disorders are common. While individuals with *1q21.1* deletions or duplications share several traits, such as borderline cognitive functioning, motor impairments, and articulation abnormalities, the duplication carriers have higher rates of ASD diagnoses and increased dimensional ASD symptom severity compared with deletion carriers. Additionally, the duplication cases had greater deficits in verbal cognitive abilities and fine motor functioning compared with familial controls with no carrier. A persistent decrease in motor abilities in carrier adults without other neurodevelopmental disorders suggests that children with *1q21.1* have a high frequency of motor impairment [54]. The phenotypic evaluation of seven new patients with interstitial microdeletions in the *8p23.2* region revealed language and speech delay and/or motor impairment, behavioural anomalies, ADHD, ASD, and dysmorphisms. However, the conventional cytogenetic analysis showed a low-level mosaicism for chromosome 8 monosomy that was not excluded by array-based comparative genomic hybridization (array-CGH) analysis, making it difficult to assess its contribution to the clinical phenotype [55].

The genetic component plays a significant role in ASD, and the most severe type of *de novo* mutation, likely gene disruptive (LGD), is closely linked with IQ, which is a phenotypic characteristic commonly associated with ASD but is not a core feature. To investigate the relationship between phenotypes and harmful *de novo* mutations in greater detail, Buja et al. conducted a study revealing that IQ and motor skills displayed distinct associations with detrimental mutations, with motor skills proving to be a more sensitive indicator of mutational severity in comparison with IQ, based on the type of mutation and target gene involved [56]. Mutations in chromodomain helicase DNA-binding protein 8 (*Chd8*), which encodes a chromatin remodeller, are a known risk factor for ASD. In a study by Kawamura et al. granule neuron progenitor (GNP)-specific deletion of *Chd8* in the cerebellum of mice was found to cause cerebellar hypoplasia and motor coordination weakness, but not ASD-like behavioural defects. This suggests that *Chd8* in cerebellar development may play a role in the pathogenesis of motor dysfunction in ASD [57]. Similarly, a study conducted by Xiao et al. focused on *BTBR T+ Itprtf/J (BTBR)* mice as a model to explore abnormal cerebellar development associated with motor impairment in autism spectrum disorder (ASD). Through their transcription analysis, they identified *TRPC* as a novel risk gene implicated in motor dysfunction in ASD [58]. Conversely, Emily et al. employed Neurexin 1α knock-out mice to investigate the behavioural outcomes and motor learning deficits. Interestingly, their findings revealed that juvenile and adult male Neurexin 1α knock-out mice exhibited social deficits and increased levels of aggression but did not display any deficits in motor learning [59]. These studies emphasize the significance of considering the developmental trajectory in mouse models used to study neurodevelopmental disorders.

Studies have also explored children with neurodevelopmental disorders with CNVs and found that coordination disorder was prevalent among them. It was also found that children’s motor coordination ability mediated ADHD symptoms, ASD clinical manifestations, and anxiety. Therefore, the abnormal development of motor coordination skills caused by CNV-induced neurodevelopmental disorders may have a cascade effect on the subsequent development of other skills, such as cognition and attention [60]. According to these findings, it is speculated that the occurrence of DCD and other neurodevelopmental disorders may be the result of the same underlying genetic causes, with different manifestations of symptoms occurring at different stages of development. This genetic pleiotropy may affect children’s brain development.

A study has examined the genetic evidence of motor impairment in neurodevelopmental disorders including ASD, ADHD, schizophrenia, and obsessive–compulsive disorder and revealed that children with motor impairment often exhibit deletions in brain-expressed genes commonly associated with other neurodevelopmental disorders [61]. Furthermore, Iman et al. conducted transcriptome profiling and found notable transcriptome similarities between ASD and schizophrenia [62]. In patients with schizophrenia, neurological soft signs (NSS), which refer to minor and subtle neurological abnormalities in sensory integration and motor performance, have consistently been observed [63].

The relationship between altered development and NSS has been reported in several studies. For instance, Peter et al. conducted a study on the British 1946 birth cohort and found that early-life motor milestones, particularly walking, may be linked to the origins of schizophrenia [64]. Birgitte et al. assessed domain-specific motor aberrations and disorder specificity among 7-year-old children with a familial risk of schizophrenia and found that motor abnormalities in children with a familial risk of schizophrenia are specific at 7 years of age with respect to fine motor function and balance [65]. Delayed motor developmental milestones, such as walking, sitting, and standing unsupported, have also been proposed as predictors of later schizophrenia [66]. Cox and Butler reviewed 200 individuals with the *15q11.2 BP1–BP2* microdeletion and identified delayed motor development, seizures/epilepsy, ASD, ADHD, and schizophrenia/paranoid psychosis as commonly observed features [67]. Another study identified *CYFIP1* as the likely gene responsible for key phenotypes of ASD and schizophrenia within the *15q11.2* region. Deficiency in *Cyfip1* leads to abnormalities in motor coordination, sensorimotor gating, and sensory perception [68]. Mohajer and Andrew investigated the offspring of parents with schizophrenia and found distinct motor and cognitive deficits, as well as abnormalities in social behaviour. Furthermore, a significant percentage of children at familial high risk for schizophrenia developed psychotic disorders in adulthood [69]. Martin et al. used a *Tubb5* mouse model, which allows for the conditional expression of the pathogenic *E401K* mutation, and found that brain-specific knock-in of the pathogenic *Tubb5 E401K* allele causes defects in motor coordination and prepulse inhibition, a phenotype associated with sensorimotor gating and considered an endophenotype for schizophrenia [70]. Motor signs have also been identified as a cluster of signs sharing substantial genetic vulnerability with schizophrenia [71,72,73].

The genetic evidence supporting the co-occurrence of DCD and ASD has been further strengthened by recent research. A comprehensive study conducted on a Japanese population uncovered significant overlap in pathogenic CNVs and biological pathways between ASD and schizophrenia, highlighting their shared genetic underpinnings [74]. Importantly, investigations have consistently shown that children with DCD exhibit a higher prevalence of CNVs compared with other neurodevelopmental disorders, and rare CNVs have been identified in this population [75]. Notably, specific mutations and small deletions in *SHANK3 (NM_033517.1)* have been associated with approximately 1% of ASD cases in children with DCD but without additional complications [76]. These compelling findings provide robust support for the notion that common genetic factors contribute to the concurrent occurrence of DCD and ASD, reinforcing the need for further exploration of shared mechanisms and pathways underlying these disorders.

## 5. Advances in Epigenetics of DCD and Its Comorbidity

Recently, researchers have shown increased interest in studying environmental effects and their interaction with genetics, particularly on epigenetic mechanisms, including DNA methylation, histone modification, and non-coding RNA regulation. In mammals, DNA methylation is a main epigenetic marker regulating genome function. It has been found that developmental environment, including maternal effects, may result in permanent changes in DNA methylation and gene expression [77]. Nevertheless, neurodevelopmental disorders, such as DCD, have limited epigenetic evidence available for reference.

Previous studies have shown that DCD may result from a combination of antenatal and postnatal environmental exposures [78,79,80] and other risk factors including preterm birth and low birth weight [79,80,81]. The multifactorial aetiology of DCD may involve both genetic and environmental risk factors during the maternal and early life periods [82]. Prematurity, such as preterm birth [11,83,84,85] and low birth weight [11,86,87], has been associated with DCD when compared with full-term delivery. The motor development of children born at different gestational ages displays variation. Specifically, preterm children exhibit comparatively poorer fine motor skills compared with full-term children [88]. Previous research indicates that white matter alterations in brain microstructure are associated with motor impairment in preterm children [89]. Moreover, an epigenome-wide study reported that 12 single-CpG- and 46 sector-based DNA methylations were linked to white matter hyperintensity burden. This finding suggests that the utilization of effective epigenomic-based biomarkers could potentially enhance health outcomes for children who are at risk of preterm birth [90]. DNA methylation is a fundamental epigenetic mechanism that monitors gene expression and determines specific transcription profiles for distinct species and cell types [91]. The addition of methyl groups to guanine bases in CpG islands by methyltransferase enzymes can impact the expression of associated genes [92]. The establishment of epigenetic patterns during development may influence gene expression, thereby increasing susceptibility to chronic diseases and influencing an individual’s health over their lifetime [93].

Various prenatal exposures, such as environmental pollutants, smoking, and alcohol have been associated with an increased risk of DCD [94,95]. A national retrospective cohort study conducted in China revealed that ambient PM2.5 exposure during pregnancy and the first 3 years of life is a significant contributor to an elevated risk of DCD [96]. The potential long-term effects of air pollution on children’s neurodevelopment are not yet fully understood. In a previous study, our team demonstrated that exposure to PM2.5 during pregnancy can induce cognitive and motor impairment in offspring mice, primarily mediated by persistent brain damage of aseptic inflammatory response and cerebellar oxidative stress [97]. Furthermore, a parallel study provided additional insight into the underlying biological mechanism of PM2.5-induced brain impairment, which is mediated by an inflammatory reaction and regulated by DNA methylation [55]. Specifically, the methylation levels of CpG sites in the promoter region of interleukin-6 were significantly reduced in mice exposed daily to concentrated ambient PM2.5 compared with those exposed to filtered air [98]. This is a hypothesis and further study is required to confirm this as a direct cause of DCD in humans.

On the other hand, the impact of maternal smoking during pregnancy on DCD has been extensively studied. It has been found that maternal smoking during the first trimester of pregnancy is significantly associated with DCD in term-born children at the age of 7 [99]; while maternal smoking during the second or third trimester has been associated with an increased risk of DCD at 8 to 9 years but not at younger ages (5–7 years) [78]. Additionally, it is important to note that, while two studies did not find any association between maternal smoking and DCD at 7–8 years [100,101] or 10 years [102], these studies had less detailed assessments of smoking (maternal report of ever smoking during pregnancy) [103,104] compared with the other two studies [78,99]. In the field of epigenetic epidemiology, the effects of maternal smoking during pregnancy on DNA methylation have been widely investigated compared with miRNA and histone modifications [105]. These investigations have revealed important epigenetic effects of maternal smoking during pregnancy that might contribute to the development of DCD in children.

Prenatal exposure to metals has been linked to neurodevelopmental outcomes, with specific metals showing negative associations with motor development [106,107,108]. While there is evidence that prenatal exposure to aluminium is associated with lower fine motor developmental quotient (DQ) and that cadmium exposure is linked to lower gross motor DQ [109], it is important to note that the causal mechanisms underlying these associations are not yet fully understood. Similarly, while lead and mercury have been identified as being neurotoxic at low doses during early development [110], further research is needed to confirm their direct impact on motor development. Nonetheless, levels of lead in maternal blood have been negatively associated with motor development [111], and postnatal mercury exposure has been found to impair fine motor function in school-age children [112]. This study also documented a relationship between plasma concentrations of polychlorinated biphenyls in 11-year-old children and poorer manual dexterity and slower fine motor speed [112]. Long-term exposure to methylmercury has been shown to damage the motor cortex in adult animals, leading to oxidative stress and a decrease in the number of neurons and astrocytes, which can impact motor skills [113]. These findings suggest that epigenetic changes in gene expression, induced by exposure to heavy metals [114], may play a critical role in shaping neurodevelopment and risk for DCD, highlighting the importance of maternal environment and other non-genetic factors in this process.

In summary, the development of neurodevelopmental disorders is influenced by a multifaceted interplay between genetic susceptibility and environmental risk factors. Prenatal exposure to certain environmental factors can increase the risk of behavioural changes in offspring, as well as reciprocal deletions and duplications in DNA, gene expression, and chromatin structure, which can significantly impact neural development. Although limited research exists on the association between DNA methylation and DCD, leveraging existing research on DCD complications and the unique characteristics of brain structure can facilitate the analysis of genome-wide methylation levels using microarray technology. This approach can identify additional factors that contribute to coordination difficulties in children with DCD. It is important to note, however, that while there is evidence linking environmental factors to motor coordination difficulties and white matter changes, there is currently no direct link between these environmental factors and specific epigenetic changes in DCD.

## 6. Conclusions and Prospects

Compared with other neurodevelopmental conditions affecting children, research on the genetics of DCD is relatively limited in both scope and quantity. Motor impairment can result from complex interactions between environmental and genetic factors. Epigenetic mechanisms, such as DNA methylation and histone modifications, may play a key role in mediating these interactions. Environmental factors, such as exposure to toxins, can cause epigenetic modifications that alter the expression of genes involved in motor function. Similarly, genetic mutations or variations can affect epigenetic marks and modify gene expression patterns in response to environmental cues. These epigenetic changes can have long-lasting effects on neural development and function, leading to motor impairment in DCD. However, while there is evidence supporting a genetic contribution to DCD, the evidence is still in its early stages, and much of the current evidence is based on studies of co-occurring conditions. Given the possibility of a shared genetic susceptibility between DCD and other neurodevelopmental disorders, future research could involve large samples of DCD-affected children as well as healthy children. Case-control and cohort studies could be conducted using whole exome sequencing and whole genome sequencing methods while taking into account the clinical manifestations and epidemiological characteristics of the disorder in the population. Genome-wide association studies (GWAS) will allow for the exploration of complex genetics and susceptibility. Analogous to other neurodevelopmental disorders, such as ADHD and ASD, the genomic architecture of DCD is likely to be highly complex, and there are currently no genes or epigenetic mechanisms directly and robustly linked to DCD. It will also be important to control potential confounding factors, such as age, in order to identify the genetic basis of DCD onset and provide insights into early diagnosis and intervention. By studying genetically homogeneous groups of individuals with reciprocal deletions and duplications who are willing to participate in long-term neuropsychometric and neuroimaging protocols, researchers will gain invaluable insight into the primary and secondary effects of genes on neurodevelopmental functions.
